# Engineering island-chain silicon nanowires via a droplet mediated Plateau-Rayleigh transformation

**DOI:** 10.1038/ncomms12836

**Published:** 2016-09-29

**Authors:** Zhaoguo Xue, Mingkun Xu, Yaolong Zhao, Jimmy Wang, Xiaofan Jiang, Linwei Yu, Junzhuan Wang, Jun Xu, Yi Shi, Kunji Chen, Pere Roca i Cabarrocas

**Affiliations:** 1 National Laboratory of Solid State Microstructures/School of Electronics Science and Engineering/Collaborative Innovation Center of Advanced Microstructures, Nanjing University, Nanjing 210093, China; 2 LPICM, CNRS, Ecole Polytechnique, Université Paris-Saclay, 91128 Palaiseau, France

## Abstract

The ability to program highly modulated morphology upon silicon nanowires (SiNWs) has been fundamental to explore new phononic and electronic functionalities. We here exploit a nanoscale locomotion of metal droplets to demonstrate a large and readily controllable morphology engineering of crystalline SiNWs, from straight ones into continuous or discrete island-chains, at temperature <350 °C. This has been accomplished via a tin (Sn) droplet mediated in-plane growth where amorphous Si thin film is consumed as precursor to produce crystalline SiNWs. Thanks to a significant interface-stretching effect, a periodic Plateau-Rayleigh instability oscillation can be stimulated in the liquid Sn droplet, and the temporal oscillation of the Sn droplets is translated faithfully, via the deformable liquid/solid deposition interface, into regular spatial modulation upon the SiNWs. Combined with a unique self-alignment and positioning capability, this new strategy could enable a rational design and single-run fabrication of a wide variety of nanowire-based optoelectronic devices.

## Abstract (web summary)

The ability to program periodic morphology into nanowires affords control over photonic and electronic transport properties. Here, the authors stimulate Plateau-Rayleigh transformations in silicon nanowires through an oscillating catalyst droplet, resulting in nanowires with island-chain morphology.

## Graphical abstract

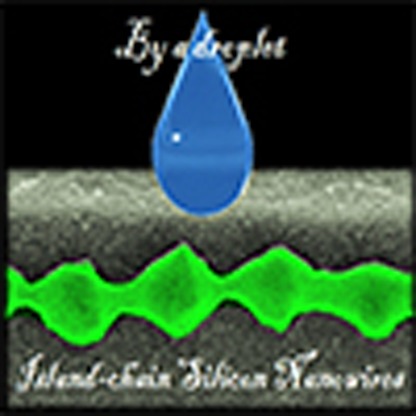

In analogy to Plateau-Rayleigh (P-R) instability in a dribbling fluid thread^1^, one-dimensional (1D) nanowires are thermally instable in view of surface Gibbs energy. If an efficient mass migration is allowed, the nanowires will tend to develop large diameter variation or just break-up into discrete spheres^2^,^3^,^4^. This kind of morphology evolution, if it happens in a controllable way, could provide an extremely powerful way to engineer the electronic, photonic and phononic transport through the silicon nanowire (SiNW) channels^5^,^6^,^7^,^8^,^9^,^10^,^11^,^12^,^13^,^14^. So far, such periodically modulated SiNW channels have been mostly manufactured by sophisticated electron beam lithography out of silicon-on-insulator wafer^11^,^15^, or by selective chemical etching into the uniform SiNWs trunks^16^,^17^. In parallel, diameter and composition-modulated superstructure are widely sought after in III–V or Ge nanowires grown via periodically controlled vapour–liquid–solid (VLS) approach^18^,^19^,^20^,^21^. Whether a simple self-organization can be tamed to engineer such sophisticated functional building blocks, ideally in a low temperature, reproducible and programmable way, is not only an intriguing fundamental challenge but also of tremendous practical importance.

It has been known that uniform-sized SiNWs experience a complete spheroidization at high temperature (>1000 °C; refs 2, 22, 23). A Plateau-Rayleigh crystal growth has been proposed, only recently, to demonstrate a discrete island-shell growth upon the sidewall of Si or Ge nanowires^3^,^4^. However, this P-R instability transformation becomes extremely inefficient at low temperature far below the melting point of Si (1414 °C for c-Si). Seeking a P-R transformation of solid c-SiNWs, at a much lower temperature and in a controllable means, requires a radically new strategy that stems from the unexplored potential of nanoscale locomotion growth.

We here report a large P-R self-transformation phenomenon during in-plane SiNW growth, mediated by low-melting-point tin (Sn) catalyst droplets that take in amorphous Si as precursor and produce crystalline in-plane SiNWs behind. Thanks to an extraordinary large interface-interaction that forces a significant stretching of the liquid catalyst droplet, which is usually unattainable in the typical VLS growth in a gaseous growth environment^24^,^25^, a periodic P-R transformation/oscillation can be stimulated in the Sn droplet and transferred to shape highly modulated SiNWs, from straight ones into continuous or discrete island-chains, all in a single-run low temperature fabrication <350 °C. In addition, this unique morphology engineering capability is accompanied with a precise self-positioning capability, and these combined are advantageous for large scale device connection and deployment.

## Results

### Fabrication of SiNWs with a-Si:H thin film precursor

**Figure 1:**
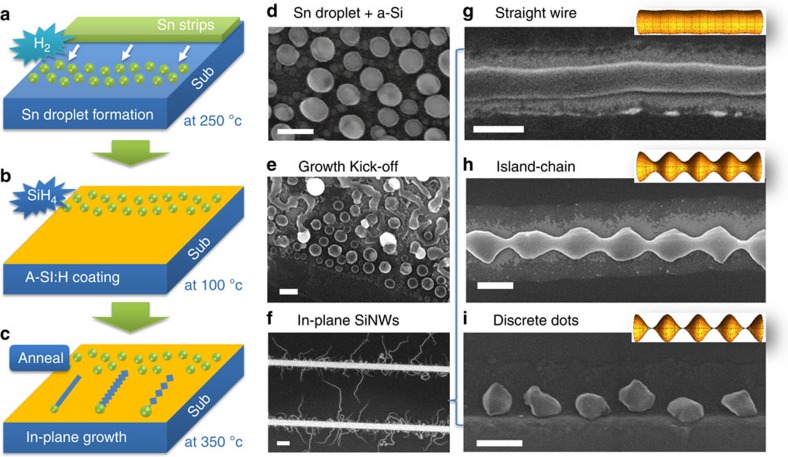
Fabrication procedure and illustration of the morphology transformation. (**a**–**c**) Illustrate the H_2_ plasma catalyst formation, a-Si:H layer coating and annealing growth steps during the Sn-catalysed in-plane growth of silicon nanowires (SiNWs), while **d** shows the scanning electron microscopy (s.e.m.) image of the Sn catalyst droplets after a-Si:H layer coating, (**e**) the *in situ* s.e.m. observation of the initial activation and growth of Sn droplets within the Sn pad stripe and (**f**) the outgrowth of in-plane SiNWs from the Sn seeding stripes; (**g**–**i**) Depicts the self-transformation of straight SiNW into continuous or discrete island-chain nanowire structure, in a way equivalent to the morphology evolution due to Plateau-Rayleigh instability. Scale bars, 400 nm in (**d**,**e**); Scale bars, 10 μm in (**f**) and Scale bars, 200 nm in (**g**–**i**).

The in-plane SiNWs are grown on SiO_2_ (300 nm)/n+-Si substrates with pre-patterned Sn stripes prepared by thermal evaporation to 20–40 nm thick. The samples are first loaded into a plasma-enhanced chemical vapour deposition (PECVD) system and treated by H_2_ plasma at 250 °C (Fig. 1a) to transform the Sn stripes into discrete Sn droplets with diameters ranging from 200 to 400 nm; Then, a thin layer of hydrogenated amorphous silicon (a-Si:H) is coated at 100 °C (Fig. 1b) to a thickness of 30 to 60 nm, as witnessed in the scanning electron microscopy (s.e.m.) image in Fig. 1d; Finally, the samples are annealed at 350 °C in a H_2_ atmosphere for 20 min, to activate the Sn liquid droplets to kick off the in-plane growth. Interestingly, as the in-plane growth requires no gas feeding and thus can be observed directly in the s.e.m. system equipped with a heating stage (see Methods for more details). In the s.e.m. image of the initial kick-off growth of the Sn droplets (Fig. 1e), the Sn droplets are identified as the bright spots that absorb a-Si:H thin film in front and produce crystalline SiNWs behind. Meanwhile, *in situ* s.e.m. images recorded during the in-plane growth of SiNWs in real-time provide the most straightforward proof of a nanoscale locomotion led by Sn droplets, as witnessed in the s.e.m. snapshots presented in Supplementary Fig. 1. However, we didnot succeed in triggering and recording an island-chain growth in *in situ* s.e.m. heating and growth, probably due to the amorphous carbon contamination on the sample surface, which is almost inevitable in our s.e.m. set-up. Usually, only bending but uniform-sized SiNWs can be obtained during an *in situ* s.e.m. heating growth. A direct comparison of the in-plane SiNWs produced via an annealing in PECVD or s.e.m. chambers are provided in Supplementary Fig. 2, as well as a more detailed discussion of this point in Supplementary Discussion.

At the end of the growth, the remnant a-Si:H layer can be selectively removed by a low temperature H_2_ plasma etching at 100 °C, while preserving very well the crystalline SiNWs^26^. In addition, in order to direct and position the in-plane SiNWs, parallel step-edge lines lying perpendicular to the Sn stripes were etched into the oxide layer to a depth of ∼180 nm before the above-mentioned fabrication procedures. A close s.e.m. image taken at 45 degree (presented in Supplementary Fig. 3) provides a better sideview of the guiding step edge configuration.

### Island-chain structure of the in-plane SiNWs

There are three distinct morphologies, as illustrated in Fig. 1c, that the Sn-mediated in-plane automation will produce in a single-run growth, which are respectively the uniform-sized straight, continuous island-chains (ic) SiNWs and the discrete-dot structures, as seen in the s.e.m. imaging in Fig. 1g–i. Particularly, the continuous island-chain SiNWs seen in Fig. 1h features an elegant sinusoidal edge-line, with a large diameter/or width modulation varying from ∼180 nm in the island to only 20–30 nm at the connecting bridges. On both sides of the SiNWs and the region, passed by the Sn droplet, a whiter region appears in the s.e.m. images as the a-Si:H layer has been absorbed. Note that, the large max/min width modulation and the rather smooth edge-lines all indicate a fundamentally different formation mechanism from the shallow corrugation observed on the sidewall of VLS-grown SiNWs^27^,^28^. Importantly, the converging trough or bridge necks are well preserved between the islands, which are indeed a prerequisite to allow efficient electric transport for electronic or thermoelectric applications.

**Figure 2:**
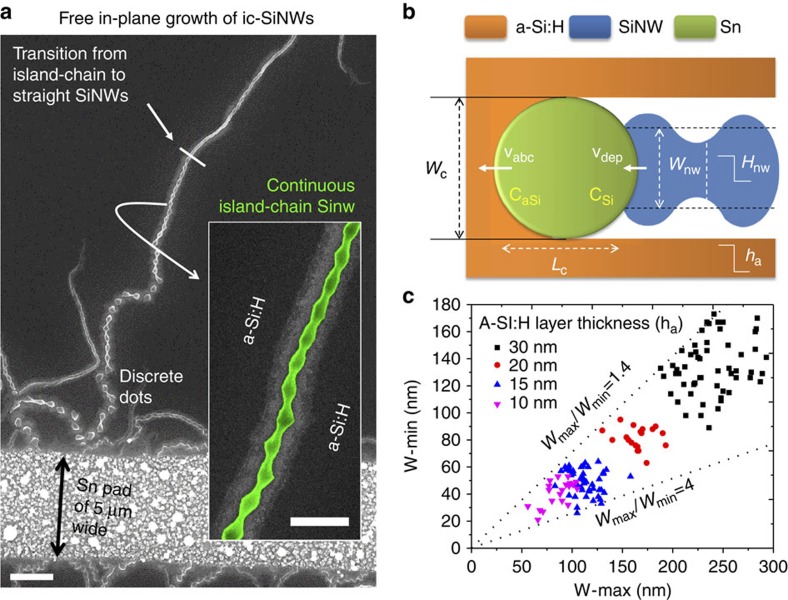
Morphology and statistics of freely grown island-chain nanowires. (**a**) Shows an enlarged view of a single island-chain silicon nanowire (ic-SiNW) grown out of the Sn pad edge, with an inset that displays a green colour-highlighted segment of the continuous island-chain structure in the middle of the SiNWs. The width of the Sn stripe is 5 μm wide. Scale bars in **a** and in the inset are 2 μm and 1 μm, respectively; (**b**) Depicts the key dimension parameters of control in the nanoscale locomotion growth system, where height of the SiNW and the a-Si:H layer are labeled as *H*_nw_ and *h*_a_, respectively; (**c**) Plots the width modulation of the in-plane island chain SiNWs, with different a-Si:H layer thickness of *h*_a_=10, 15, 20 and 30 nm

Remarkably, though the outgrowth of the in-plane SiNW can reach as long as >50 μm, as seen in the s.e.m. image in Fig. 1f, a closer scrutiny of single SiNWs reveal, however, three growth stages with clear morphology transition from discrete to continuous island-chain, and to uniform-sized SiNWs, as witnessed in Fig. 2a. Departing from the Sn pad edge, the relatively thick SiNWs are found to be composed of discrete islands/or grains, which then gradually merge with the neighbours into a continuous island-chain structure, as seen in the inset of Fig. 2a, when the in-plane growth proceed further. Later on, even the width modulation amplitude diminishes gradually, and eventually switch into a uniform-sized SiNWs. For instance, this transit from the island-chain growth into uniform-sized ones has been marked by a dashed line in Fig. 2a. Meanwhile, it is also noteworthy that such an island-chain growth are only observed for the Sn-catalysed growth of SiNWs, while the indium-catalysed in-plane growth leads to usually uniform-sized SiNWs^29^,^30^. In addition, plotting the max/min width ratio variations for the continuous ic-SiNWs with different initial a-Si:H layer thicknesses *h*_a_ in Fig. 2c shows that (1) the mean width of the in-plane growth of ic-SiNW scales up linearly with the increase of the a-Si:H layer thickness; and (2) the max/min width ratios share a common linear up-bound, delineated by *r*_d_∼140%, while the maximum is obtained for the thinner ones (with proportional thinner *h*_a_ as well) with a ratio beyond *r*_d_ >400%.

In parallel, reference island-chain (ic) SiNW samples with shorter annealing growth times of 10 min and 2 min are also prepared, with corresponding s.e.m. images shown in Supplementary Fig. 4. Compared with the ic-SiNWs out of 20-min annealing growth as seen in Fig. 2a, we found that the Sn droplets were almost exhausted after the first 10 min annealing growth, leaving behind ic-SiNWs with similar lengths and morphology features. Meanwhile, the Sn catalyst droplets can be observed after only 2-min annealing growth, as shown in Supplementary Fig. 4c,d, where the island-chain structures are produced immediately after the leading Sn catalyst droplets, without the final stage of uniform-sized SiNW. These observations indicate that the island-chain structures are not produced via direct P-R morphology transformation of the uniform SiNWs (after they were produced by the Sn catalyst droplet), rather the large diameter modulation are directly shaped or forged by the leading Sn droplets, as a consequence of large oscillation dynamics during the Sn droplet's in-plane movement, as will be discussed in more details later.

It is also noteworthy that, according to the atomic force microscopy profile reconstruction and the cross-section analysis of a continuous island-chain SiNW segment presented in Supplementary Fig. 5, the in-plane SiNWs typically have a larger width than their height, *W*_nw_>*H*_nw_. This feature can be understood as a consequence of a surface-constraint in-plane growth, where the precursor thin film supply is limited on the lower substrate surface, which is also in a strong contrast to that in gas-feeding VLS growth environment (usually leads to a round cross section).

### Self-positioned guided growth of the ic-SiNWs

**Figure 3:**
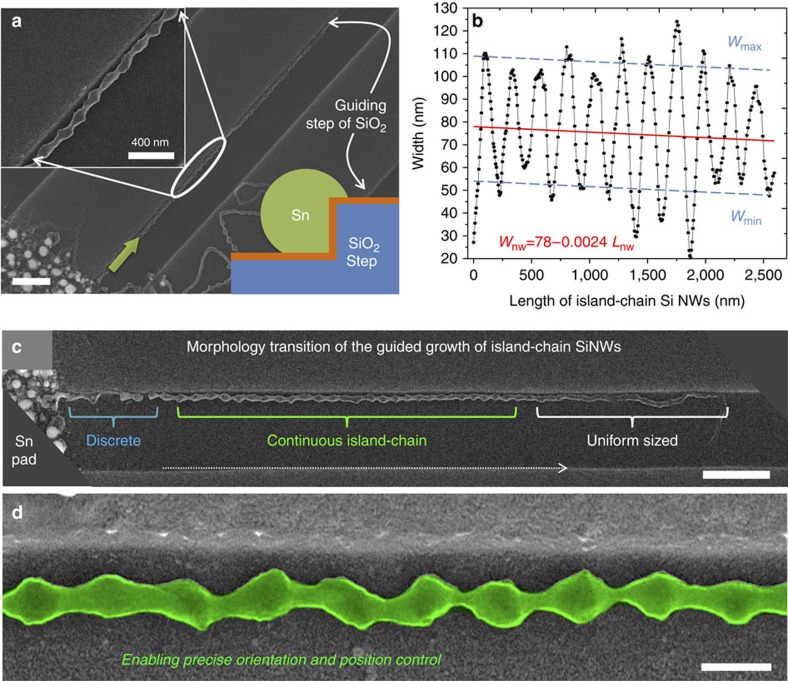
Morphology and statistics of guided growth island-chain nanowires. (**a**) Show the s.e.m. images of the guided growth of island-chain silicon nanowires (ic-SiNWs) along the step-edges formed by etching into the SiO_2_ substrate, with a configuration as depicted by the inset; (**b**) Summarizes the width variation, as well linear fittings of the average, minimum and maximum width of the guided growth of SiNW, along the length of the ic-SiNW segment marked in green in **d**,**c** provides an overview of an ic-SiNWs, starting from the Sn pad edge on the left, which experiences a series of morphology transition, during the guided in-plane growth, from discrete to continuous island-chains and then to uniform-sized ones. Scale bars, 1 μm in (**a**); scale bars, 1 μm in (**c**) and scale bars, 200 nm in (**d**).

More excitingly, the growth of ic-SiNWs by itself can be directed into precise direction and locations, with the aid of simple step edges formed by etching into the SiO_2_ dielectric layer and lying perpendicular to the Sn stripes, as seen in Fig. 3a. A schematic illustration of the guided growth along step edge has been placed in the bottom-right inset, depicting a cross-section view of the guided growth SiNWs with an extra contact line on the vertical sidewall that can help to attract the catalyst droplet to move along. This self-positioning or guided growth is indeed a unique capability of the in-plane growth of SiNWs^26^,^31^, which is important to deploy SiNW-based functionalities over large scale, without the need of any extra nano-manipulation. A statistics of the width variation along the length of a guided ic-SiNW is plotted in Fig. 3b, with the corresponding s.e.m. image marked in green shown in Fig. 3d. A linear fitting of the width evolution in Fig. 3b reveals a slightly decreasing trend of the mean width of 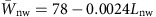 along the growth direction.

### Structural analysis

**Figure 4:**
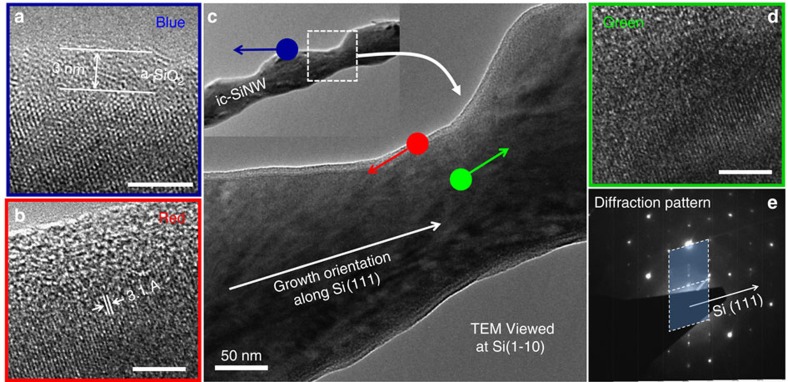
Structural analysis of an island-chain nanowire segment. (**a**,**b**,**d**) Show the high-resolution transmission electron microscopy lattice images taken three different locations at the island maximum (marked by the blue spot), the edge (red) and the center (green) of the converging trough neck, as indicated in **c**. According to the electron diffraction pattern recorded in the center in **e**, the growth direction of this ic-SiNW is determined to be along Si[111] direction. Scale bars, 5 nm in (**a**); scale bars, 5 nm in (**b**); scale bars, 5 nm in (**d**).

Despite of a large geometry modulation, a high crystallinity has been confirmed within the ic-SiNWs, as witnessed in Fig. 4a–d, where a series of high-resolution transmission electron microscopy (HR-TEM) characterizations are presented. Surrounding by a thin native oxide (a-SiO_2_) layer of ∼3 nm thick on the sidewall, the HR-TEM lattice images, taken at three different locations, indicated a coherent Si lattice throughout the ic-SiNWs, for example, in the regions marked by the blue spot in Fig. 4a at the edge of the island maximum, and the green and the red ones at the edge and the center of a converging trough in Fig. 4b–d, respectively. Also, for this specific ic-SiNW segment, the growth direction is identified to be along Si[111] orientation, as determined by the electron diffraction pattern presented in Fig. 4e. This is somehow deviated from the conventional Si[211] growth direction of the in-plane SiNWs led by indium droplets^26^,^32^.

## Discussion

In order to understand this unique self-automated growth dynamics, we come to formulate the in-plane growth with a set of key parametric dimensions as indicated in Fig. 2b. In general, a higher Gibbs energy of Si atoms in the amorphous state is the key driving force for the Sn catalyst droplet to absorb Si atoms continuously from the front a-Si:H/Sn interface and precipitate c-SiNWs at the rear Sn/SiNW deposition interface. Due to a higher Gibbs energy seen by the Si atoms in the amorphous status compared with that in crystalline phase, δ*E*∼0.11 to 0.15 eV (refs 33, 34), the equilibrium concentration of Si in the liquid Sn droplet at the front a-Si:H/Sn interface (*C*_aSi_, in contact to an a-Si:H medium) is higher than that of the rear Sn/c-Si interface (C_0_, in contact to c-SiNW) with a relation of *C*_aSi_=*e*^*δE*/*kT*^*C*_0_. This equilibrium concentration difference will drive a diffusion flux of the dissolved Si atoms front the front interface towards the rear deposition interface, and thus establishing a supersaturation of C_Si_>*C*_0_ that leading to an axial growth rate of


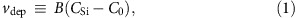


where *B* is assumed to be a constant pre-factor. In the meantime, the front absorption interface advance and move on, with a speed mainly limited by the diffusion transport of Si atoms over the length of the Sn catalyst droplet *L*_c_, as


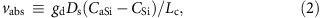


where *D*_s_ is the diffusion constant of Si atoms in Sn droplet, and *g*_d_ the geometric correction factor accounting for the asymmetric width of the front and rear interfaces.

Actually, a distinctive aspect of the in-plane growth of SiNW is that the front Sn/a-Si:H and the rear SiNW/Sn boundaries are both solid-liquid interfaces, and both of them can exert significant forces upon the liquid catalyst droplet sandwiched in between, which is in strong contrast to the situation of conventional VLS growth where a soft gaseous precursor environment. During the in-plane growth, the droplet always tends to presume a spherical shape while its length is always constrained by the relative positions of the two bounded interfaces, that is, the absorption front interface and the deposition interface contacting the SiNW end. As a consequence, the temporal variation of *L*_c_ is determined by the relative speeds of the front absorption and the rear deposition interfaces, as


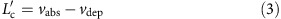


Meanwhile, the evolution of the dissolved Si atom concentration at the rear Sn/c-SiNW interface is governed by the competition between the absorption and deposition terms with


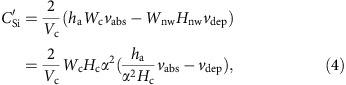


where *W*_c_ and *W*_nw_ are the widths of the absorption and the deposition interfaces, as defined in Fig. 2b, 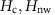 and *h*_a_ the heights of the Sn droplet, the SiNW and the a-Si:H layer, respectively. The width and the height of the SiNWs are always proportional to the dimensions of the Sn droplet, with *W*_nw_=*αW*_c_ and *H*_nw_=α*H*_c_.

**Figure 5:**
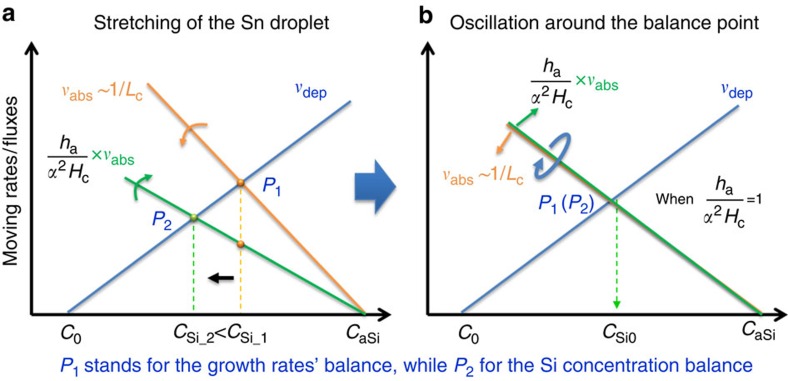
Growth balance point evolution and oscillation during in-plane growth. The blue (or orange) line in **a** represents the evolution trend of the rear deposition (the front absorption) rate as a function of the Si concentration (*C*_Si_), while the green one stands for the product of the absorption flux with a geometry-dependent pre-factor of *h*_a_/*α*^2^*H*_c_; (**b**) Illustrates the coincided balance points achieved when the pre-factor term *h*_a_/*α*^2^*H*_c_=1, and their periodic oscillation around the balance points.

According to equation (1), the moving rate of the rear deposition interface can be represented by an blue line in Fig. 5a that increases linearly with higher Si atom concentration of *C*_Si_ dissolved in the Sn droplet, while the moving rate of the front absorption interface is plotted as the orange line that decreases, according to equation (2), with the increase of *C*_Si_ and drops to zero when *C*_Si_=*C*_aSi_. These two lines cross at a balance condition point *P*_1_ sought by equation (3) to arrive 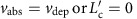. However, this will not always guarantee a balanced state for the evolution of *C*_Si_ in equation (4), unless the pre-factor before *v*_abs_ within the parenthesis of equation (4) equals unity 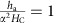. In case of 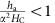 with a crossing point between the green absorption line and the blue deposition line at *P*_2_, to the left of *P*_1_, the deposition term in equation (4) outweighs the absorption and thus the Si concentration will decrease from *C*_Si1_→*C*_Si2_ to approach *P*_2_. When this continues, the front interface will pick up speed and the rear interface will slow down a little bit. As a consequence, the Sn droplet is forced to deform, being elongated in order to bring down the slope of the absorption line, which is ∼1/*L*_c_ according to equation (2). In the meantime, a longer *L*_c_ causes a thinner *H*_c_ (and *W*_c_ alike), and enlarges the factor of 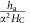 as well, to meet the inclining *v*_abs_ line. In principle, if the initial catalyst diameter is 
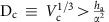
, the Sn droplet will be driven into a stretching state with longer *L*_c_ and shorter *H*_c_ and *W*_c_, otherwise the opposite will take place.

**Figure 6:**
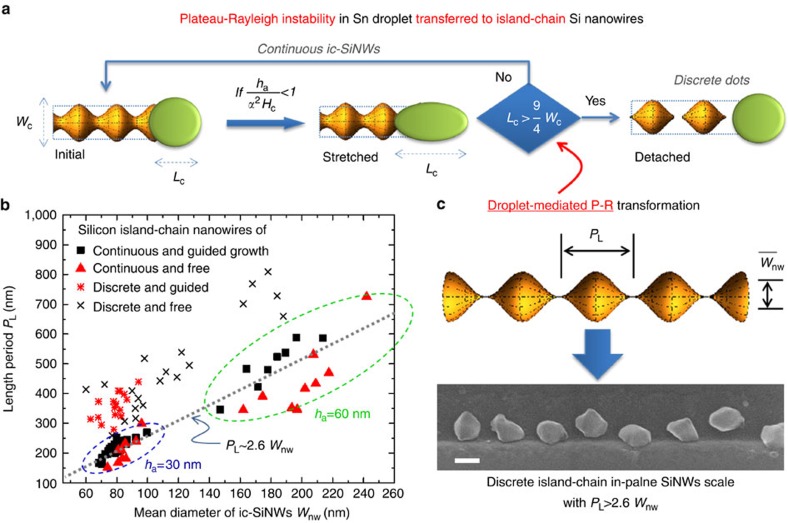
The formation of continuous or discrete island-chain nanowires. (**a**) Plateau-Rayleigh instability of the Sn catalyst droplets, under stretching, is recorded upon the as-produced SiNWs, leads to continuous or discrete island-chain geometry depending on whether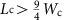, the criterion for P-R transformation; (**b**) Presents a statistics of the continuous or discrete island-chain SiNWs, with or without guiding step edges, grown with two a-Si:H layer thicknesses of *h*_a_=30 and 60 nm, respectively, as marked by the blue and the green dashed circles; (**c**) Provides the typical s.e.m. of the discrete Si dots structure. Scale bar, 200 nm.

At the end of the evolution in Fig. 5a, the Sn droplet will be stretched to satisfy 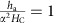, and thus the two balance points (*P*_1_ and *P*_2_) will coincide as depicted in Fig. 5b. Normally, this balance will be maintained if it is stable against external perturbation. This is, however, not always the case, because in view of the Gibbs surface energy, an elongated geometry is energetically unfavorable. Based merely on a surface tension consideration, the stretched Sn droplet, as illustrated in Fig. 6a, can be viewed as a segment of a continuous liquid rod/thread, where a Plateau-Rayleigh instability/transformation will happen whenever the length of the free droplet segment (*L*_c_) is approaching 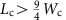, where *W*_c_ is the width of the catalyst droplet (see Supplementary Fig. 6 and Supplementary Discussion for the derivation). Note that, this self-transformation, arising from P-R instability will cause a gradual shrinkage of the rear deposition interface, and thus shape a narrowing neck/trough of the as-produced SiNWs, as *W*_nw_=*αW*_c_.

Depending on how fast this nanoscale locomotion system can re-establish the balance, as discussed above, a thinner *H*_c_ has a counter effect to speed up the advancement of the rear deposition interface to catch up the front interface, there are two possible eventual geometries, as depicted schematically in Fig. 6a. In the first situation, if the counter mechanism can respond quickly enough, to reverse the elongation of the Sn droplet, continuous island-chain morphology will be produced; On the contrary, if the P-R transform proceed to the end, the Sn droplet can eventually detach from the pointed Si grain behind, in order to restore a spherical shape. Then, a new round of nucleation and growth will initiate and repeat, leaving a chain of discrete Si dots along the moving course, as witnessed for instance in Fig. 6c. This is indeed a remarkably new type of growth dynamics, where a low-melting-point catalyst droplet is confined by a pair of moving and interacting interfaces, and is forced to deform to a large extent that causes an automated P-R instability transformation. In turn, this droplet oscillating dynamics is faithfully recorded by the as-produced SiNWs, enabling an unprecedented morphology control of the functional 1D building blocks.

In order to gain deeper insights of this periodic growth of ic-SiNWs, we write down a very general form of the overall surface Gibbs energy, for the sake of brevity but without loss of generality, as 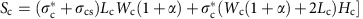, where 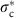
is the mean surface energy density of Sn catalyst droplet (that also absorbs the contributions from Sn/SiNW and Sn/a-Si:H interfaces), while *σ*_cs_ stands for the interfacial energy density of the Sn/substrate interface. Under a constant volume constraint of *V*_c_≡*W*_c_(1+*α*)*L*_c_*H*_c_/2 and let *L*_c_ determined by the relative positions of the two interfaces, the liquid catalyst droplet is allowed to adjust *W*_c_, in order to minimize the S_c_ by


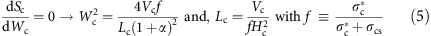


Combining equation (3) and equation (4), the second temporal derivative of *L*_c_ is written as


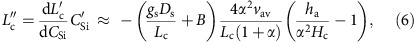


where the speeds of the two interfaces are replaced by their mean velocity 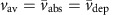 at the equilibrium balance point. Assuming a periodic form of 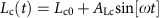, where *A*_Lc_ is the amplitude of oscillation, as well *W*_c_(*t*)=*W*_c0_+*A*_Wc_sin[*ωt*+*π*] and *H*_c_(*t*)=*H*_c0_+*A*_Hc_sin[*ωt*+*π*]. Note that, a phase shifting of *π* is imposed as 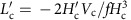 according to equation (5). Inserting these into equation (6), with the derivation detailed in Supplementary Discussion, the first criterion to stimulate a periodic dynamics is


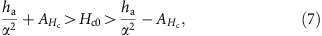


where *H*_c0_ is proportional to the initial size of the Sn catalyst droplet, as *H*_c0_∼*V*_c_^1/3^. Specifically, the second inequality in equation (7) set the first constraint for the ic-SiNW growth that


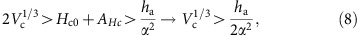


which tells that the Sn catalyst droplet has to be large enough compared to the a-Si:H layer thickness of *h*_a_ in order to trigger an oscillating growth dynamics. Otherwise, the oscillation dynamics will quickly damp away, leading to uniform-sized SiNWs as seen in Fig. 1g. This also explains why at the end of the in-plane growth, when the Sn droplet shrinks, an ‘island-chain to uniform-sized' transition will happen as observed in Fig. 2a and Fig. 3c. Actually, approaching to this transition point, the oscillation amplitude *A*_Hc_ will also diminish to *A*_Hc_≪*H*_c0_, which refines this criterion further to 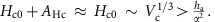 This indicates that, for a larger Sn droplet compared to a given a-Si:H thickness, in order to re-establish the growth balance condition as specified in Fig. 5b of 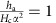, the Sn droplet will tend to develop a ‘stretching' status, which is exactly the key to trigger a P-R instability engineering of ic-SiNWs, as discussed above.

On the other hand, the first inequality in equation (7) requires that 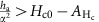. This implies that, when the Sn droplet is large with 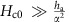, the size oscillation amplitude also has to increase proportionally. To the extreme of very large amplitude, that's *A*_Hc_∼*H*_c0_, the as-produced SiNWs will become simply discontinuous, as seen in Fig. 1i, though a precise description of this large-deformation dynamics falls beyond the capability of this qualitative analytic model. In short, to the lower end of Sn droplet size, only uniform-sized in-plane SiNWs will be produced, while to the higher end, the ic-SiNWs will tend to become discontinuous. This finding is indeed crucial, as controlling the relative size of the Sn droplet with respect to the given a-Si:H layer thickness (or 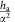) can provide a powerful and convenient means to program the morphology of the Sn-catalysed in-plane SiNWs.

Furthermore, expanding the last term in equation (6) to the first order around 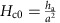, that is, 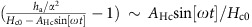, we have


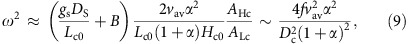


where the first term on the right is proportional to 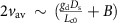 and *D*_c_∼*V*_c_^1/3^. Thus, the length period *P*_L_ of the island-chain nanowires can be deduced to be


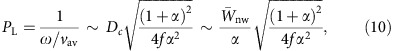


where the mean width (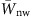) of the island-chain SiNWs is proportional to the cross section dimension of the Sn droplet 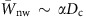. equation (10) implies that the length period of the ic-SiNWs (*P*_L_) should be proportional to the mean with of ic-SiNWs. And this relation has been examined in Fig. 6b, where the length period *P*_L_ of the ic-SiNWs, grown freely or being guided along step-edge represented by the red triangles or the black circles, respectively, have been plotted against their mean width 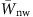. Discrete ic-SiNWs are usually found with a larger length period above the scaling low of continuous ones. Interestingly, though with two different a-Si:H coating layer thicknesses of *h*_a_=30 nm or 60 nm, the groups of continuous ic-SiNWs points split into two distinct groups as delineated by two dashed circles in blue and green, their distribution follows clearly a scaling trend of *P*_L_∼2.6*W*_nw_, which agrees fairly well with what we expect of the lower-bound of 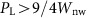 for a Plateau-Rayleigh instability dominated self-transformation.

In other words, the island-chain nanowires are cast out of periodically vibrating Sn catalyst droplets under stretching, where the deformable deposition interface serves as a kinetics channel to translate the periodic droplet oscillation into spatial periodicity encoded upon the as produced SiNWs. It is important to emphasize that this self-automated island-chain modulation is fully programmable by means of controlling the a-Si:H layer thickness with respect to the initial catalyst size control. Combined with a unique self-positioning capability, these new findings will pave the way for future rational fabrication and practical device deployment.

In summary, we here demonstrate a unique Plateau-Rayleigh morphology engineering of 1D SiNWs mediated by a nanoscale locomotion of tin (Sn) catalyst droplets. Particularly, we have been able to fabricate well-defined and controllable island-chain SiNW structures via a low temperature (<350 °C) thin film deposition, and have shown that the island-chain SiNWs can be easily positioned along step-edges that enable precise location control. These results emphasize a unique morphology tailoring capability of such a nanoscale locomotion that will eventually enable a straightforward manufacturing of more sophisticated, as well more functional, nanostructures and devices.

## Methods

### SiNW fabrication

The in-plane SiNWs are fabricated a conventional PECVD system on n+-Si wafer substrate with a thermal SiO_2_ layer of 300 nm thick. Parallel Sn stripes of of ∼40 nm thick and 2 μm wide were defined by lithography patterning, thermal evaporation and lift-off. The samples are first clean by ultrasonic prior to loading into the PECVD chamber for H_2_ plasma treatment at 250 °C for 5 min; Then, a thin layer of hydrogenated amorphous silicon (a-Si:H) is deposited at 100 °C by pure silane plasma, with a flow rate of 10 standard cubic centimetre per minute (SCCM) and radio frequency (RF) power of 2W, to 30 nm ∼ 60 nm; After that, the substrate temperature is raised again to 350 °C and a H_2_ atmosphere annealing is carried out for 20 min, with a chamber pressure of 133 Pa. During this course, the in-plane growth of SiNWs are activated mediated by the surface-moving Sn liquid droplets; Finally, the remnant a-Si:H layer is removed by a low temperature H_2_ plasma etching at 100 °C.

### *In situ* s.e.m. annealing and observation

The samples are first prepared with the same fabrication procedure in PECVD system as detailed above, except the final annealing step. Then, the samples are unloaded and transferred to s.e.m. (Zeiss Sigma) system, with an air-exposure period <2 min. Within the s.e.m. chamber, equipped with a heating stage (Kammrath & Weiss, Heating Module 800 °C), the substrate temperature is increased to 330 °C for 5 min to activate the catalyst droplets, and then ramped up to 350 °C for in-plane SiNW growth observation. During this course, the chamber vacuum is maintained at <5 × 10^−3^ Pascal.

### Guiding step-edge preparation

For the guided in-plane growth of SiNWs, step edge lines are defined by lithography and subsequence reactive ion etching (RIE) etching into the underlying 300 nm oxide layer to a depth of ∼180 nm. The spacing between the guiding edge-lines are around 20 μm. In the next step, Sn stripes were prepared, with the above-mentioned procedure, but lying perpendicular to the guiding edge lines.

### Data availability

All relevant data are available from the corresponding authors on request.

## Additional information

**How to cite this article:** Xue, Z. *et al*. Engineering island-chain silicon nanowires via a droplet mediated Plateau-Rayleigh transformation. *Nat. Commun.* 7:12836 doi: 10.1038/ncomms12836 (2016).

## Supplementary Material

Supplementary Information Supplementary Figures 1-6 and Supplementary Discussion.
