# Supplementary material for: Engineering island-chain silicon nanowires via a droplet mediated Plateau-Rayleigh transformation
Source: Nat Commun. 2016 Sep 29;7:12836. doi: 10.1038/ncomms12836 (PMC5056411; doi:10.1038/ncomms12836)
Supplement: Supplementary Information — Supplementary Figures 1-6 and Supplementary Discussion. [file ncomms12836-s1.pdf]

Supplementary Figures

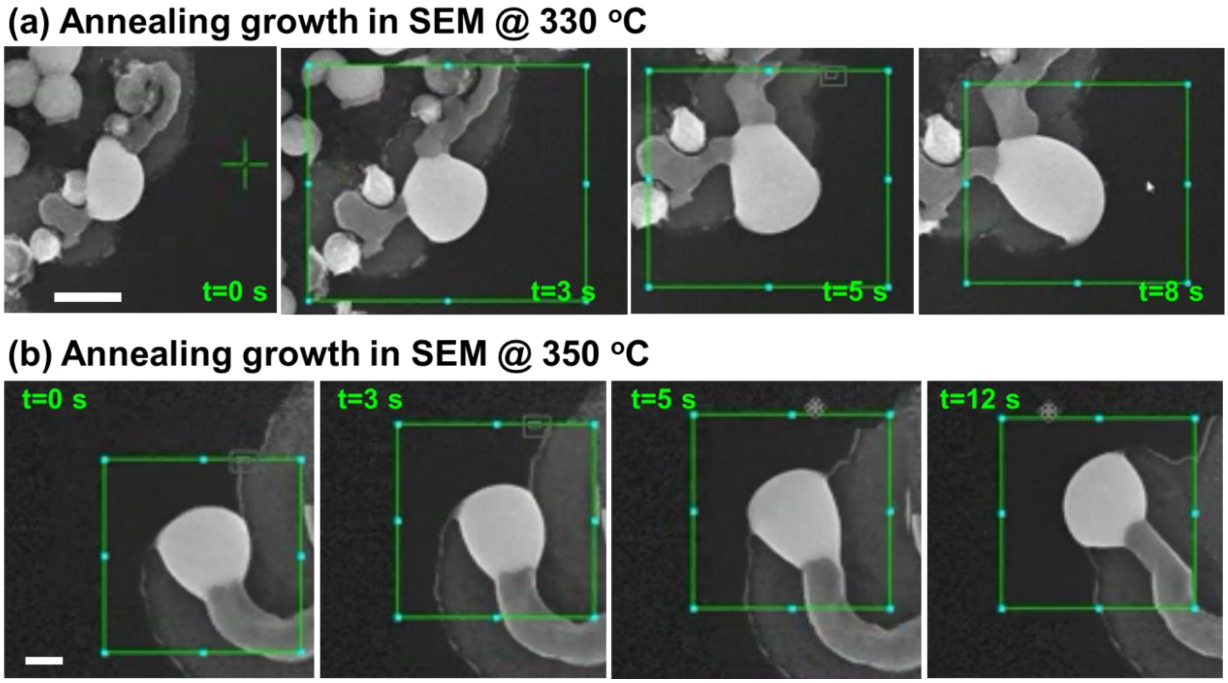

Supplementary Figure 1: *In-situ* scanning electron microscopy imaging of the in-plane SiNWs growth. (a) and (b) show the snapshots of Sn droplets activated to grow during *in situ* scanning electron microscopy (SEM) annealing at 330 °C and 350 °C, respectively, when they depart from the catalyst pad edge with an obvious 'stretching' deformation with a longer length and narrower width. The scale bars in (a) and (b) are 500 nm and 200 nm, respectively. Note that, this significant geometry distortion of the leading catalyst droplet, confined by the front absorption and the rear deposition interfaces, is a unique aspect during an in-plane growth of silicon nanowires (SiNWs), which is also in a strong contrast to the typical vapor-liquid-solid (VLS) growth happens within a soft environment.

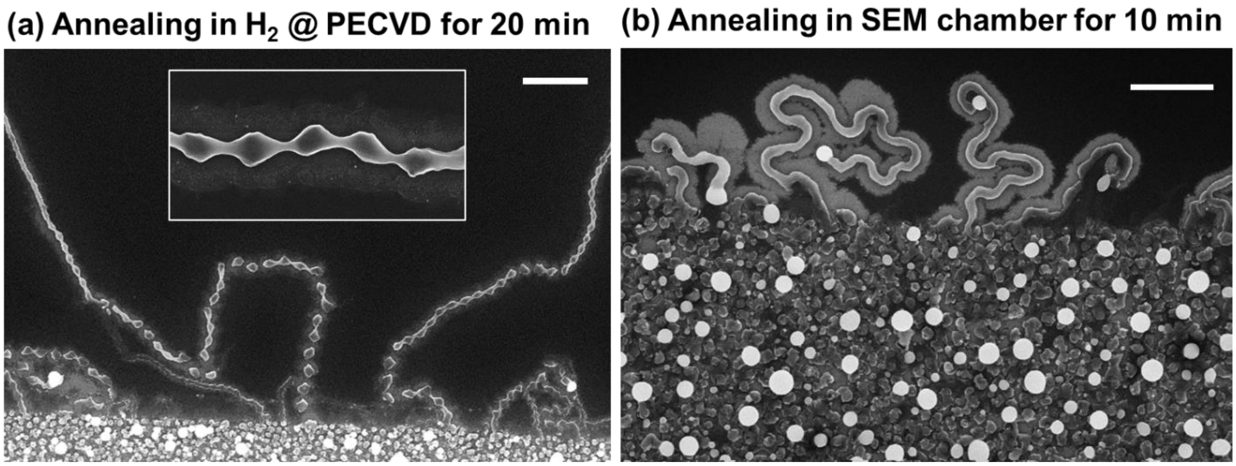

Supplementary Figure 2: Different nanowire morphology from PECVD or SEM chamber annealing. A comparison of the in-plane silicon nanowires (SiNWs) obtained via an annealing growth in (a) H_2 ambient in PECVD chamber for 20 min, or (b) in the SEM chamber for around 10 min, with the same growth control parameters and at basically the same growth temperature. The scale bars are 2 μm and 1 μm, respectively.

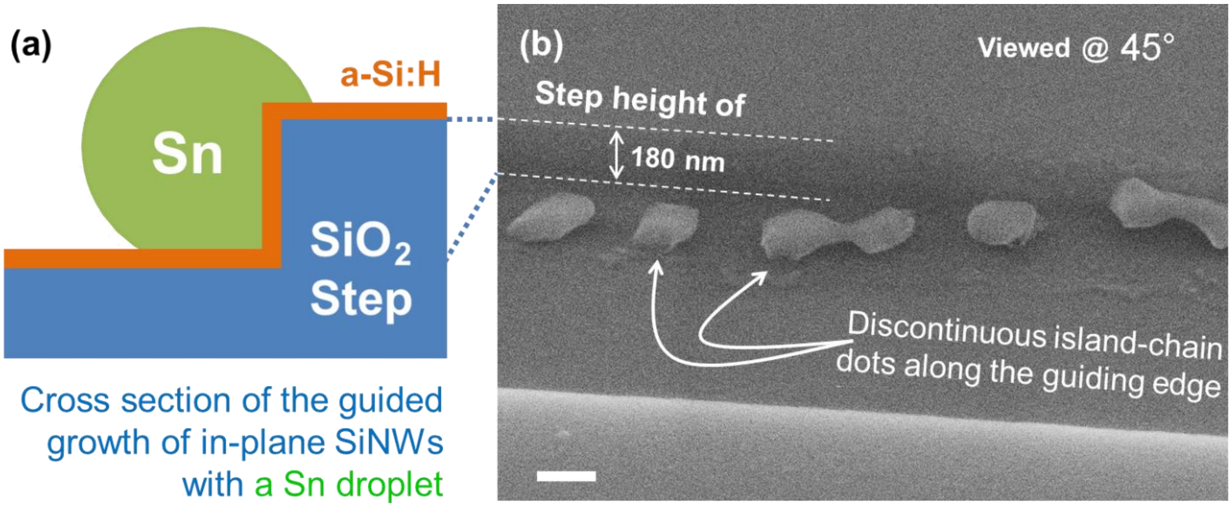

Supplementary Figure 3: Illustration and SEM side-view of the guided and discontinuous island-chain nanowires. (a) Schematic illustration of the cross-section of the guided growth of in-plane Si nanowires (SiNWs) led by a Sn droplet that forms two absorption contact lines on the bottom and the side-wall surface, both coated with a-Si:H layer; (b) side-view SEM image of a guided and discontinuous SiNW along the guided edge. The scale bar is 200 nm.

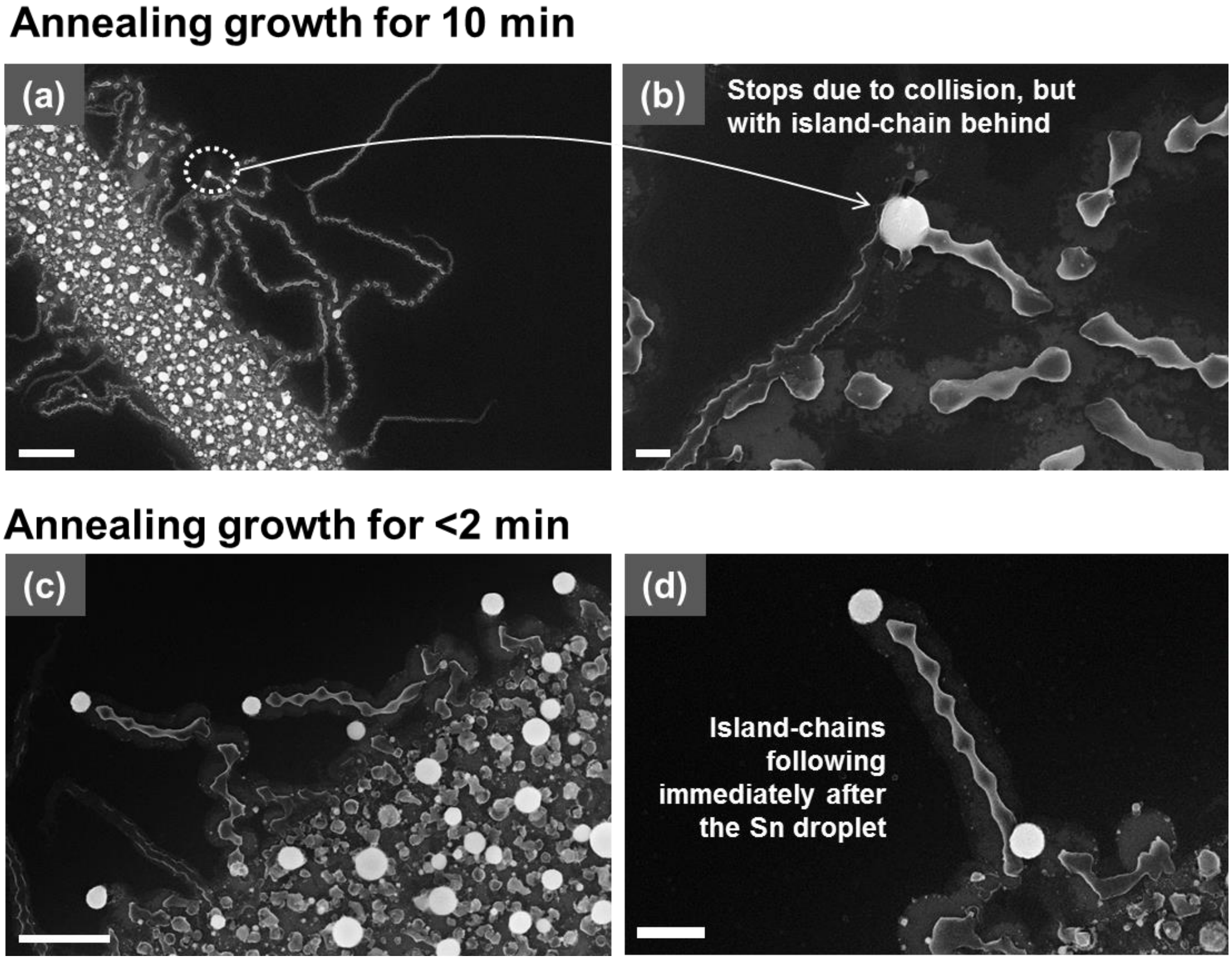

Supplementary Figure 4: In-plane nanowires after different annealing growth durations. (a) and (b) The scanning electron microscopy (SEM) images of the in-plane island-chain silicon nanowires (SiNWs) after annealing growth for 10 min in the PECVD Chamber in H_2 ambient; (c)

and (d) The SEM images of the SiNWs after a much shorter annealing growth time < 2min. The scale bars in (a)-(d) are 2 μm, 200 nm, 1 μm and 400 nm, respectively

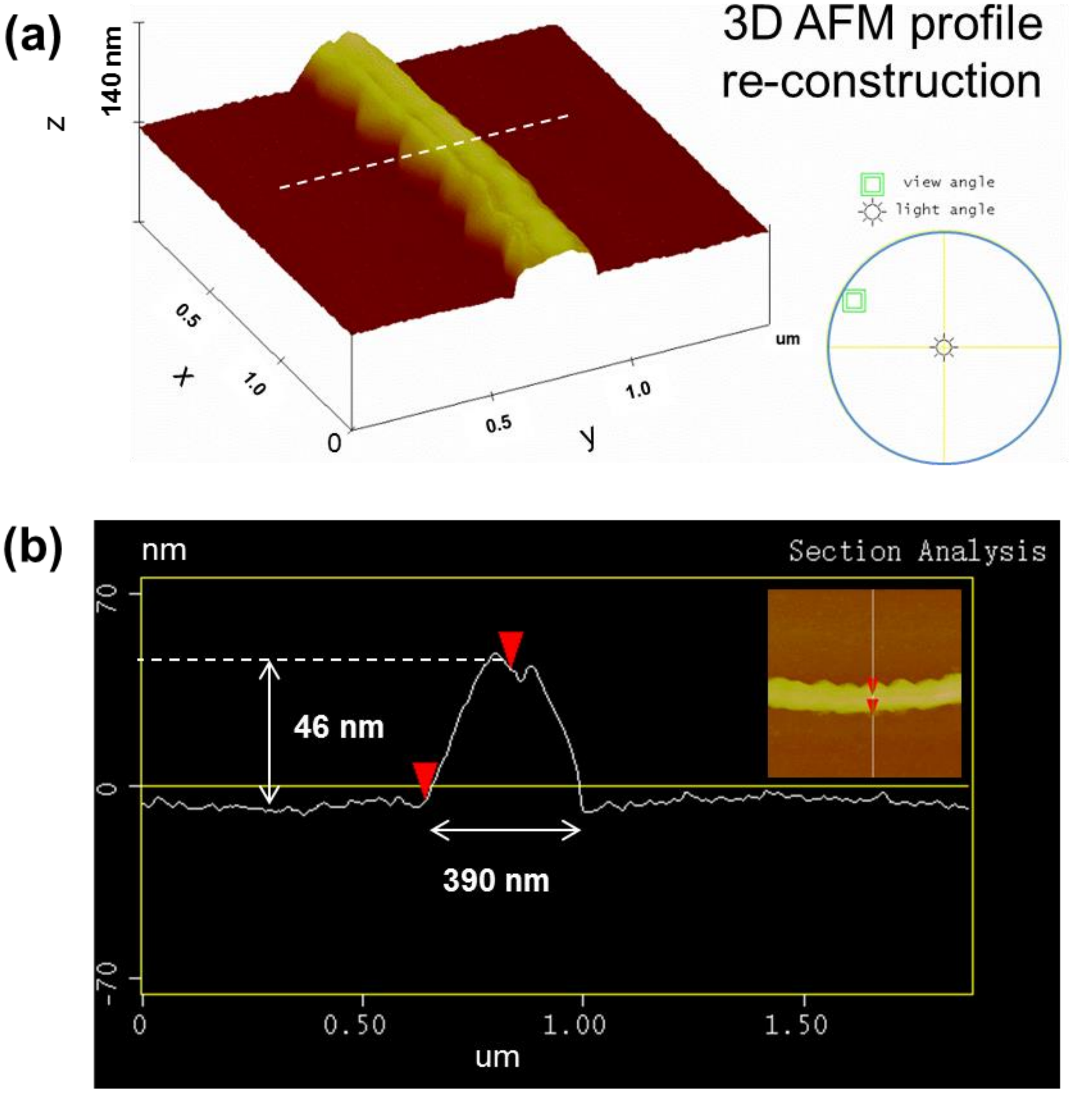

Supplementary Figure 5: Atomic force microscopy characterization of the island-chain nanowires. (a) and (b) show the 3D atomic force microscopy (AFM) profile reconstruction and

the cross-section analysis of a continuous island-chain SiNW segment, respectively. The width varies from 200 nm to 390 nm with a height less than 50 nm. This geometry feature can be understood as a consequence of a surface-constraint in-plane growth, where the precursor thin film supply is always limited on the lower substrate surface. Note that discrete or continuous island-chain SiNWs with radical diameter variations are not ideal for AFM scanning imaging. Therefore, we choose only the island-chain SiNWs with only a relatively small diameter modulation.

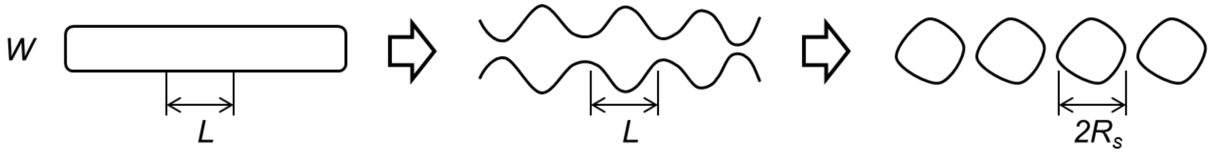

Supplementary Figure 6: Plateau-Rayleigh instability transformation of uniform wire segment into discrete dots. Schematic illustration of the evolution of a uniform-sized nanowire into discrete island-chains, driven by the trend to minimize the surface energy.

Supplementary Discussion

Different nanowire morphologies from PECVD chamber or SEM chamber annealing.

Note that, according to our experiments, an in-plane growth of the island-chain SiNWs cannot be triggered during *in situ* scanning electron microscopy (SEM) heating growth. Even with exactly the same growth parameters (including the Sn layer thickness, the a-Si layer coating and all the other plasma deposition conditions), the island-chain SiNW structure can be very well

reproduced in PECVD system, but only wavy SiNWs will be produced during an *in situ* SEM heating growth, see Supplementary Figure 2 for comparison. The effect of surface oxidation inevitable during the sample transferring can be excluded, as we have tried to minimize the air-exposure time to several minutes, and confirmed that the island-chain growth can be re-initiated by re-introducing the samples (after half hour air-exposure) back to the PECVD system and annealing them in H_2 ambient or just in vacuum. Actually, the hydrogen passivation on the a-Si:H surface is known to last >15 min against oxidation in ambient, usually long enough for sample transferring between systems.

Derivation of Plateau-Rayleigh instability transformation of uniform wire segment into discrete dots.

Considering the transformation of a segment of straight and uniform-sized wire of W wide and L long, as illustrated in the Supplementary Figure 6, an equal-volume sphere has a radial of

$$\pi \frac{W^2}{4} L = \frac{4}{3}\pi R_s^3 \rightarrow R_s = \left(\frac{3}{16} W^2 L\right)^{1/3} . \quad (1)$$

The transformation becomes energetically favorable if the exposed surface energy on the sidewall of straight nanowire is larger than that of an equal-volume sphere, that is,

$$\pi W L \sigma > 4\pi R_s^2 \sigma = 4\pi\sigma \left(\frac{3}{16} W^2 L\right)^{2/3} \rightarrow L > \frac{9}{4} W, \quad (2)$$

where σ is the surface energy density of the nanowire system. In other words, if there is a free segment of uniform-sized nanowire with $L > \frac{9}{4} W$, a Plateau-Rayleigh self-transformation will

tend to take place, as long as mass redistribution is allowed and efficient (as is the case for the liquid Sn catalyst droplet, but not for the solid silicon nanowire at low temperature). Meanwhile, it is important to note that, this kind of Plateau-Rayleigh transformation is not expected for the uniform-sized SiNWs after they were produced by the Sn droplets, as such a low annealing growth temperature.

Derivation of the parametric window for triggering an island-chain Si nanowire growth.

Assuming a general temporal evolution form for the oscillating catalyst droplet as follows

$$L_{\mathrm{c}}(t) = L_{\mathrm{c0}} + A_{\mathrm{Lc}}\ Sin[\omega t], \tag{3-1}$$

$$W_{\mathrm{c}}(t) = W_{\mathrm{c0}} + A_{\mathrm{Wc}} Sin[\omega t + \pi] \tag{3-2}$$

$$H_{\mathrm{c}}(t) = H_{\mathrm{c0}} + A_{\mathrm{Hc}} Sin[\omega t + \pi] \tag{3-3}$$

where a phase shifting of π has been imposed because $L_c' = -2H_c' V_c / H_c^3$. Therefore, we have

$$-\omega^2 A_{\mathrm{Lc}} Sin[\omega t] = -M\left(\frac{h_{\mathrm{a}}/\alpha^2}{H_{\mathrm{c0}} + A_{\mathrm{Hc}} Sin[\omega t + \pi]} - 1\right) \tag{4}$$

$$\rightarrow\ Sin[\omega t] = \frac{\mathrm{M}}{\omega^2 \mathrm{A_{Lc}}}\left(\frac{h_{\mathrm{a}}/\alpha^2}{H_{\mathrm{c0}} + A_{\mathrm{Hc}} Sin[\omega t + \pi]} - 1\right) \tag{5}$$

where $\mathrm{M} \equiv \left(\frac{g_{\mathrm{s}} D_{\mathrm{S}}}{L_{\mathrm{c}}} + B\right) \frac{4\alpha^2\ v_{\mathrm{av}}}{L_{\mathrm{c}}\ (1+\alpha)}$ and the pre-factor term is always positive, as $L_{\mathrm{c}} > 0$, the sign of the part on the right high side is all determined by the term within the brackets. In order to allow an oscillating behavior, Supplementary Equation 5 tells that we should have, at least,

$$\frac{h_{\mathrm{a}}/\alpha^2}{H_{\mathrm{c0}} + A_{\mathrm{Hc}}} - 1 < 0 \ \text{ and } \ \frac{h_{\mathrm{a}}/\alpha^2}{H_{\mathrm{c0}} - A_{\mathrm{Hc}}} - 1 > 0, \tag{6}$$

which translates into a parametric window written as

$$\frac{h_{\mathrm{a}}}{\alpha^2} + A_{\mathrm{H_c}} > H_{\mathrm{c0}} > \frac{h_{\mathrm{a}}}{\alpha^2} - A_{H_{\mathrm{c}}}, \quad (7)$$

where H_{c0} is proportional to the initial size of the Sn catalyst droplet, as $H_{\mathrm{c0}} \sim V_{\mathrm{c}}^{1/3}$.
